# Irradiation of Neurons with High-Energy Charged Particles: An *In Silico* Modeling Approach

**DOI:** 10.1371/journal.pcbi.1004428

**Published:** 2015-08-07

**Authors:** Murat Alp, Vipan K. Parihar, Charles L. Limoli, Francis A. Cucinotta

**Affiliations:** 1 Department of Health Physics and Diagnostic Sciences, University of Nevada, Las Vegas, Las Vegas, Nevada, United States of America; 2 Department of Radiation Oncology, University of California, Irvine, Irvine, California, United States of America; University of California at Berkeley, UNITED STATES

## Abstract

In this work, a stochastic computational model of microscopic energy deposition events is used to study for the first time damage to irradiated neuronal cells of the mouse hippocampus. An extensive library of radiation tracks for different particle types is created to score energy deposition in small voxels and volume segments describing a neuron’s morphology that later are sampled for given particle fluence or dose. Methods included the construction of *in silico* mouse hippocampal granule cells from neuromorpho.org with spine and filopodia segments stochastically distributed along the dendritic branches. The model is tested with high-energy ^56^Fe, ^12^C, and ^1^H particles and electrons. Results indicate that the tree-like structure of the neuronal morphology and the microscopic dose deposition of distinct particles may lead to different outcomes when cellular injury is assessed, leading to differences in structural damage for the same absorbed dose. The significance of the microscopic dose in neuron components is to introduce specific local and global modes of cellular injury that likely contribute to spine, filopodia, and dendrite pruning, impacting cognition and possibly the collapse of the neuron. Results show that the heterogeneity of heavy particle tracks at low doses, compared to the more uniform dose distribution of electrons, juxtaposed with neuron morphology make it necessary to model the spatial dose painting for specific neuronal components. Going forward, this work can directly support the development of biophysical models of the modifications of spine and dendritic morphology observed after low dose charged particle irradiation by providing accurate descriptions of the underlying physical insults to complex neuron structures at the nano-meter scale.

## Author Summary

Changes to cognition and other central nervous system (CNS) effects following charged particle exposures are of concern for medical patients undergoing radiation treatment in cancer therapy and for cosmic ray exposures to astronauts during space travel. Neuronal cell injury initiated by ionizing energy deposition involves a cascade of physico-chemical and patho-physiological pathways that are poorly understood. Neurons have diverse structural compartmental geometries, including the cell soma, dendrites, and spines. The intricate network of cellular connections requires the study of radiation damage to neuronal morphological features in understanding cellular injury and possible changes in cognitive function. This paper for the first time describes the spatial dependence of a particle’s microscopic dose deposition events on a detailed neuron structure. Heavy ions including iron, carbon and hydrogen particles, and energetic electrons that are common in space radiation exposures are considered. The computational model covers the stochastics of the generation of energy deposition events, delivery of particle beams and possible encounter schemes of microscopic dose and neuronal morphology. Results from this *in silico* study can be used to consider experimental studies in order to understand structural and functional neuronal damage following irradiation.

## Introduction

Neuronal cells show great diversity in morphology and are highly organized within the animal brain [1]. In general, neurons are differentiated cells with a tree-like structure including the soma which contains the cell nucleus, numerous dendritic branches emanating from the soma unidirectionally or from multiple poles, and a single axon. Numerous spines protruding from dendrites make synaptic connections with other spines and dendrites located on other neurons [2]. Neurons are excitable cells with a plethora of specialized ionic membrane channels that participate actively and passively in changes of membrane potential to transduce, relay and integrate information within the neurocircuitry of the nervous system [3]. Neuronal cells are also dynamic, as branches may extend and immature filopodia seek new connections to make synapses with cellular cues [4,5].

The hippocampus plays an important role in learning, consolidation of memory and retrieval of information [6,7]. The dentate gyrus (DG) in the hippocampus shows great similarity in cellular morphology and neurocircuitry across mammalian species [8]. The principal neurons in the dentate gyrus are dentate granule cells (DGCs) [9,10]. The DGC dendrites extend perpendicularly to the granule cell layer, into the overlying molecular layer where they receive synaptic connections mainly from the entorhinal cortex via the perforant pathway. DGCs are mostly monopolar neurons as dendrites emerge from apical portion of the cell body. Mossy fibers are the axons of the granule cells extending from the basal level and making synapses with the pyramidal cells of CA3 region of the hippocampus [11,12].

Investigations into ionizing radiation damage to cognition over a large range of absorbed doses (<0.1 Gy to 10’s of Gy where the Gray (Gy) is the unit of absorbed dose defined as 1 Joule/kg)) and for distinct radiation qualities, including for high linear energy transfer (LET) radiation, is of growing interest [13–15]. The use of protons and carbon beams in so-called Hadron therapy for cancer treatment has increased by more than an order of magnitude in this century with a growing number of facilities in the U.S., Japan and Europe [16,17]. Continued interest in space exploration where astronauts are exposed to cosmic rays including protons, iron and other heavy ions has led to research studies in mice and rats demonstrating cognitive detriments at doses below 0.5 Gy [18–20]. In addition, as treatment of brain cancers with conventional radiation (electrons and photons) has improved, longer patient survival and efforts to understand and mitigate cognitive changes is currently an important focus [21,22].

Neuronal injury leading to cellular death is likely initiated by structural damage to the tree-like dendritic arborization of neurons, which generally extend hundreds of microns from the soma [23–27]. In addition, cell signaling and the induction of neuro-inflammation or other detrimental changes will follow from the initial energy deposition events, which vary with the type of radiation as well as dose or particle fluence. In the past, energy deposition within neuronal cells and its influence on these processes has not been studied.

The primary goal of the present work is to develop a model of microscopic energy deposition (ED) events in neurons from different radiation qualities and doses. Neuronal morphological data of a DGC was chosen as an example to explore the effects of ionizing radiation [28], both to validate the model with accumulating data on DGCs [13,19,23] and make testable predictions for future studies. The simulations described are for particles in the so-called track segment mode where a particle undergoes a negligible decrease in velocity as it traverses a path-length through a volume, which are representative of space radiation exposures and normal tissue damage in therapy. Calculations for particles near the Bragg peak where LET reaches a maximum and particles are close to stopping important within the tumor volume in therapy are not discussed. Because high charge (Z) and energy (E) (HZE) particles create numerous secondary electrons denoted as δ-rays, calculations for a typical electron energy of 0.5 MeV are also described. This energy is also representative of electrons produced by a gamma-ray or X-ray sources. The *in silico* approach developed can be expanded to other types of neurons and regions in the central nervous system (CNS) to quantify energy deposition at microscopic scale for a given charged particle type and dose.

## Materials and Methods

### Irradiation library

Energy deposition (ED) events from particle tracks are simulated with the RITRACKS software [29,30] (available from http://spaceradiation.usra.edu/irModels). RITRACKS is a Monte-Carlo based computer model that calculates the positions of the ED events due to ionization and excitation as a particle traverses a media assumed to be water. The stochastic simulation environment for given charged particle with an initial energy, propagation direction (e.g. along the *z*-axis) and entrance point, e.g. (0,0,0) creates ED events at varying positions for the initial particle and the created secondary electrons denoted as δ-rays, which are correlated with the particles path. Energy deposition events are lumped within a predetermined voxel size [30,31]. The total ED in a voxel and its coordinates are an output value from the simulation. For a predetermined track length (*L*_*Track*_), voxel ED values and their coordinates are calculated, which includes contributions from the primary particle track and δ-rays. This stochastic process is repeated a large number of times using Monte-Carlo techniques with the same initial conditions to create a library of histories for a particles ED events. In this study, all the voxel sizes and track lengths are taken as 20 nm^3^ and 20 μm, respectively. Particle kinetic energies are expressed in units of MeV for protons and electrons, and MeV per atomic mass unit (u) for HZE particles. We considered simulations of ^56^Fe at 600 MeV/u, ^12^C at 300 MeV/u and ^1^H at 250 MeV particles to create a library of 5,000, 10,000 and 20,000 histories, respectively that are used for calculations of energy deposition in the neuron structures considered. For the 20 μm track length, the initial energies are reduced by (0.01, 0.007, 0.003)% respectively at the end of the track. The average LET values calculated over all the histories are (172.4, 12.9, 0.4) keV/μm.

HZE particles liberate numerous electrons through ionization in passage through a media such as water, and some of these electrons denoted as δ-rays deposit their energy outside a defined test volume where neurons are bounded. Because the range of δ-rays exceeds 0.1 cm for the energies considered, the appropriate volume to use to sample the particle track was a focus of numerical simulations as described below and in the S1 Text. We also considered 500 keV electrons, which is a typical energy of the δ-rays produced by HZE particles. Results do not depend critically on the starting electron energy for energies above about 50 keV. To obtain ‘nearly uniform energy’ electrons, a library of 20,000 histories was created starting from the same initial energy and propagation direction by following the dose deposition events from 500 keV to 490 keV.

### Simulating neuron morphology in the dentate gyrus

The neuron morphology of a hippocampal granule cell of C57BL6/Trk.T1 deficient mouse was acquired from publicly accessible repository neuromorpho.org (ID: NMO_07656) [32,33]. The soma and dendrites are represented as cylindrical segments with axes points and radii. The NMO_07656 has 16 branches with total branch length (*L*_*DN*_) of 1392 μm, total soma and dendrite volume of 1129 μm^3^ and 1559 μm^3^, respectively (adds to total volume of 2688 μm^3^). The overall measure of (width, height, depth) is (169, 180, 69) μm that is given a diagonal extension to 256 μm. These geometric measures are in agreement with the compiled 70 mouse hippocampal granule cells authored by Vuksic and Lee at neuromorpho.org website resulting in (mean±SD) for total volume (5631±1720 μm^3^), branch number (25±5), total branch length (2161±384 μm) and diagonal extension (279±33 μm) [32,33].

### Spine and filopodia statistics

**Fig 1:**
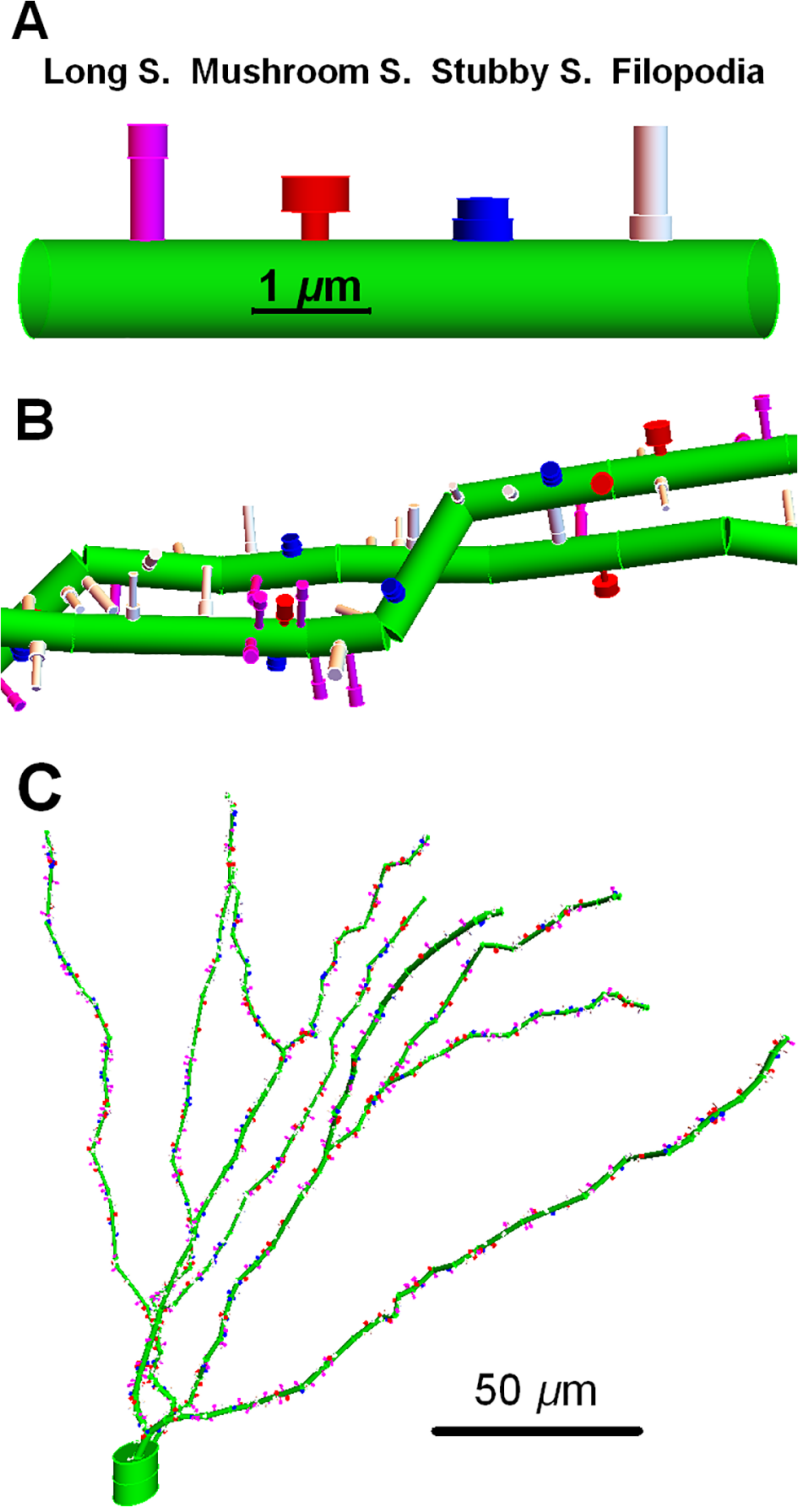
The test neuron; a dentate granule cell including spines and filopodia that are randomly distributed on the dendrites. Filopodia and three types of spines are randomly distributed along the dendrites. In color code (A), dendritic segment is green, with spines (Ss) tall (magenta), mushroom (red), stubby (blue), and filopodia (white), which are located on the surface of the dendritic segment. (B) A statistical realization of segments of dendritic branches with spines and filopodia. (C) a snap shot of the test neuron including the soma (two large green cylinders), the dendrites, spines and filopodia are shown with the scale bar.

The reconstructed anatomical data do not include the spine and filopodia information of the mouse DGCs at the neuromorpho.org repository. Different tissue staining techniques (Golgi-Cox, horseradish peroxidase) and green/yellow fluorescent expressing transgenic mouse (e.g., Thy1-GFP) with high magnification confocal images provide the statistics of total number of spines, filopodia per unit length (10 μm). We utilized the published data for Thy1 GFP mouse hippocampal granule cell linear spine density statistics (*D*_*SN*_) by the publications Parihar and Limoli [19] and Parihar et al. [13], and value of *D*_*SN*_ = 4.3±0.4 per 10 μm. Spines are also categorized depending on their morphology as tall, mushroom and stubby spines, Fig 1. Their ratio is adapted from the same publications. In addition, not matured invaginations; filopodia linear density, *D*_*FN*_, of 4.3±0.4 per 10 μm was adapted from the same publications.

### Stochastic distribution of spines and filopodia along the dendrites

The total dendritic length (*L*_*DN*_ μm) was found by adding the height of dendritic cylindrical segment lengths by keeping track of segment number and base to ceiling directional information. A random number for total number of spines (*N*_*ST*_) was found from a normal distribution with given mean and standard deviation (*N*_*ST*_ = (*L*_*ND*_/10) × *NormalDist*(*D*_*SN*_). The total number of *N*_*ST*_ uniform random points was located on the corresponding segments and distance from the base of the segment. The same procedure was applied to locate total number of filopodia (*N*_*FT*_) on the dendritic segments. Then, the distance between all spine and filopodium points are calculated and if there was any pair distance less than 0.5 μm, a new random location on the dendrite was found for one of the pairs. This procedure was repeated until all the spines and filopodia are separated with given distance criteria. Random axial directions of all spines and filopodia pointing perpendicular to the segment surface were found by drawing uniform random points between *0* to *2π*. Finally, spines are registered as tall, mushroom and stubby spines by choosing random numbers from a normal distribution with given mean and standard deviation for two types and assigning the remaining registration for the third kind by keeping the *N*_*ST*_ value constant.

Spines and filopodia were represented as two cylinder segments with overlapping axes directions and different diameter and axes heights (**Fig 1**). These cylinders are named spine/filopodia *base* segment protruding from surface of the dendrite and *top* segment on the *base* cylinder. Spine morphology is categorized by long, mushroom and stubby with different heights and diameter. The ratio of these three types of spines is adapted from [13,19]. Segment heights and diameter are similar for the filopodia and long spines. These geometric factors including different spine ratios are presented in **Table 1**. The filopodia is a functionally separate category. A stochastic realization of the spine/filopodia distribution on the dendrites was run once and kept constant thorough this paper and these numbers are also presented in **Table 1**.

**Table 1:** Spines and filopodia cylindrical axes heights and diameter values.

Spines & Filopodia	Ratio	Base-Head Height±SD (μm)	Base-Neck Height±SD (μm)	Top-Head Diameter±SD (μm)	Top-Neck Diameter±SD (μm)	Numbers used in this paper
Long	0.38	0.4±0.07	1±0.12	0.35±0.06	0.3±0.06	250
Mushroom	0.33	0.42±0.08	0.35±0.06	0.6±0.1	0.24±0.06	213
Stubby	0.29	0.25±0.04	0.25±0.04	0.46±0.04	0.54±0.04	168
Filopodia	1	1.1±0.1	0.3±0.06	0.3±0.05	0.38±0.05	577

### Scoring of hits between particle beam and neuronal segments

A sample particle beam of length *L*_*Sample*_ at ‘nearly uniform energy’ can be constructed by randomly selecting *n*_*sample*_ (*ceiling*(*L*_*Sample*_/*L*_*Track*_)) histories of pre-simulated tracks from a corresponding library. First, sample histories can be translated along the propagation direction and data can be stitched together to create a beam that traverses the complete volume. Then, further translations and rotations operations can be applied on the beam for any beam, target coincidence event detection. In addition, multiple processors in a parallel computation can also take advantage of operating on individual *L*_*track*_ histories of a reconstructed beam instead of creating a new beam with many voxel values. The scoring process determines the coincidence of ED in voxels from a particle beam within the neuronal volume. A trial of the scoring library for any given charged particle utilizes the histories of the irradiation library to construct a beam that targets the sample neuron. The randomness between each trial includes the random construction of ED events of a beam and relative random orientation of the beam and the neuron.

**Fig 2:**
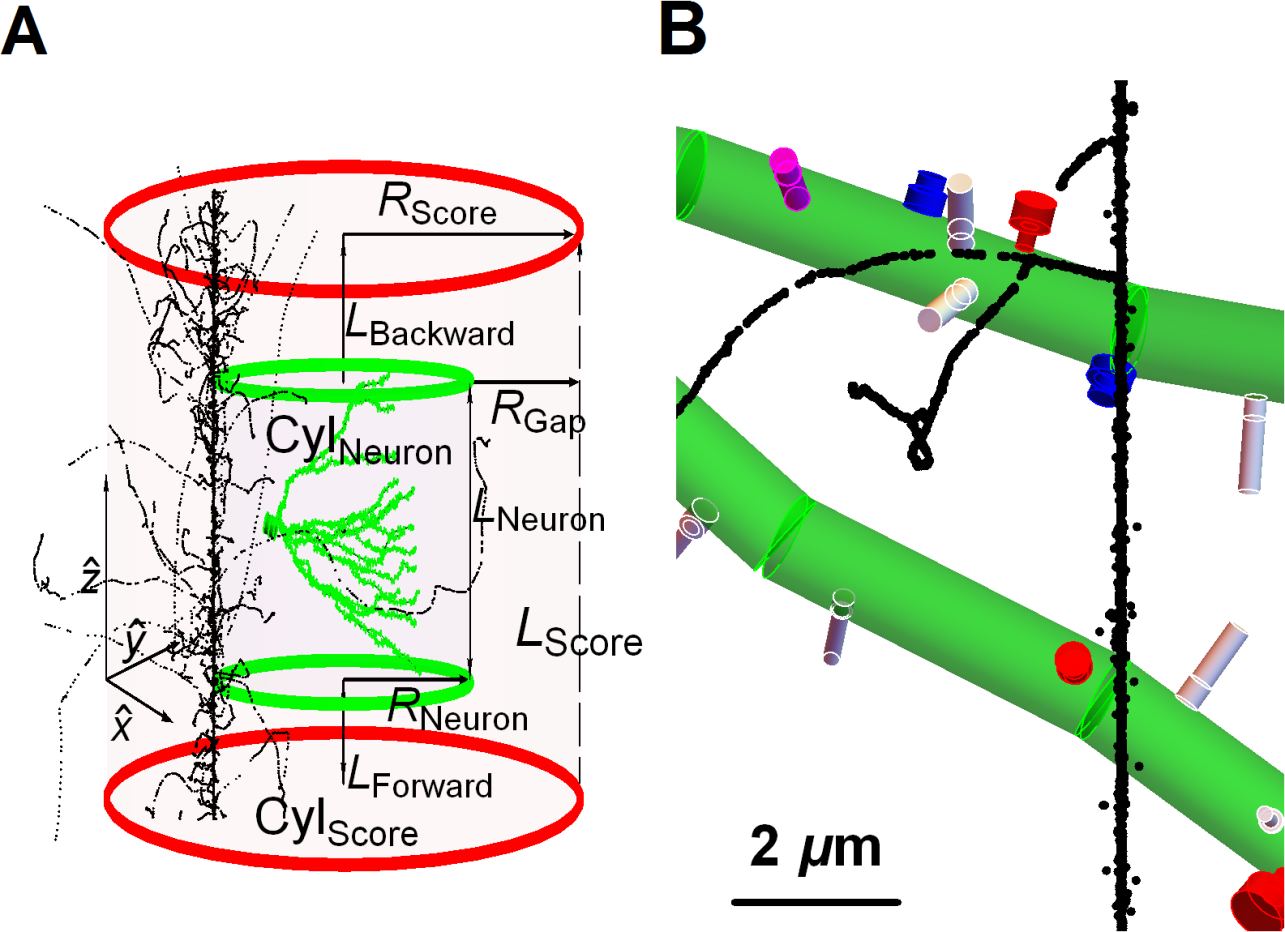
Volumes of neuronal compartments, particle beam and scoring. (A) At each trial, a random particle beam enters at a random point on *xy*-plane and propagates along the +*z*-direction. The neuron is bounded by the volume *Cyl*_*Neuron*_ and embedded on a larger scoring volume *Cyl*_*Score*_. *L*_*Score*_ is the height of the *Cyl*_*Score*_ and randomly selected 20 μm tracks from the irradiation library are lined up to create a particle beam equal or larger than *L*_*Score*_. The constant distances between the cylinders *L*_*Forward*_, *L*_*Backward*_ and *R*_*Gap*_ contribute to the electronic equilibrium in *Cyl*_*Neuron*_. *L*_*Neuron*_ is the maximum extension of the neuron along the *z*-axis and changes at each trial with random orientation of the neuron. Particle fluence is calculated as 1/*A*_*Score*_ where $A_{Score}=\pi R_{Score}^{2}$. If there are one or more coincidence events, then the trial is recorded as a ‘hit’. For clarity, only one out of twenty voxels are shown for a ^56^Fe beam at 600 MeV/u in (A) and 20 nm^3^ voxels are shown much larger than their actual size on the panels. (B) A close up view of ^12^C at 300 MeV/u which is grazing the dendritic branches.

The geometry of a beam and a neuron configuration in **Fig 2** is organized as follows: The scoring geometry contains two cylinders with overlapping cylinder axes along the *z*-axes with different axes length and radii. The larger scoring cylinder (*Cyl*_*Score*_) has the base at (*x*,*y*,0) and axis length of *L*_*Score*_ that is equal to *L*_*Score*,*i*_ = *L*_*Forward*_ + *L*_*Neuron*,*i*_ + *L*_*Backward*_. The radius, *R*_*Score*_ of the *Cyl*_*Score*_ is given as *R*_*Score*_ = *R*_*Gap*_ + *R*_*Neuron*_. The lengths, *L*_*Forward*_, *L*_*Backward*_, *R*_*Gap*_ are constants for any beam type versus neuron simulations and discussed in detail in the S1 Text. *R*_*Neuron*_ is the test neuron constant and *L*_*Neuron*,*i*_ varies between each trial *i* as discussed below. Scoring is done for each beam by testing the coordinates of each voxel and segment boundaries of the neuronal cylinders if the voxel is inside of any segments of the neuron. If there are one or more coincidence events, then the trial is recorded as a ‘hit’. **Fig 2** illustrates that a large number of ED events are concentrated close to the particles path main, while the number of scattered electrons per unit distance increases with LET of the particles and can extend far from the particles location.

The neuron is bounded by the smaller cylinder (*Cyl*_*Neuron*_) that has the maximum diameter (radius of *R*_*Neuron*_) and axis length (*L*_*Max*,*Neuron*_) of 256 μm that is the diagonal extension of the neuron. The base of the *Cyl*_*Neuron*_ is located at (*x*, *y*, *L*_*Forward*_) but has variable axis length *L*_*Neuron*,*i*_ (≤*L*_*Max*,*Neuron*_) at each trial *i* for computational purposes as discussed below. A unique geometric center is found for the neuronal tree and this center is situated along the longitudinal axis of the *Cyl*_*Neuron*_, as well as the *Cyl*_*Score*_. The neuron makes random rotations around its geometric center at each trial *i* and the coordinates of any dendritic cylindrical segments that have the farthest and the closest distances to the *z* = 0 plane are found. This range is specified as *L*_*Neuron*,*i*_ and the cylindrical coordinates of rotated neuron is translated along the axis such that the geometric center coincides with (0, 0, *L*_*Forward*_ + (*L*_*Neuron*,*i*_/2)) point.

A relative random orientation of a particle beam and the neuron is set by propagating the beam along the +*z*-axis, parallel to the neuronal cylinder axis at each trial and randomly rotating the neuron as described above. These translation and rotation information are recorded during the trial as the coordinates and energies of coincidence events are replaced back to the initial neuron configuration by back rotation and translation.

**Fig 3:**
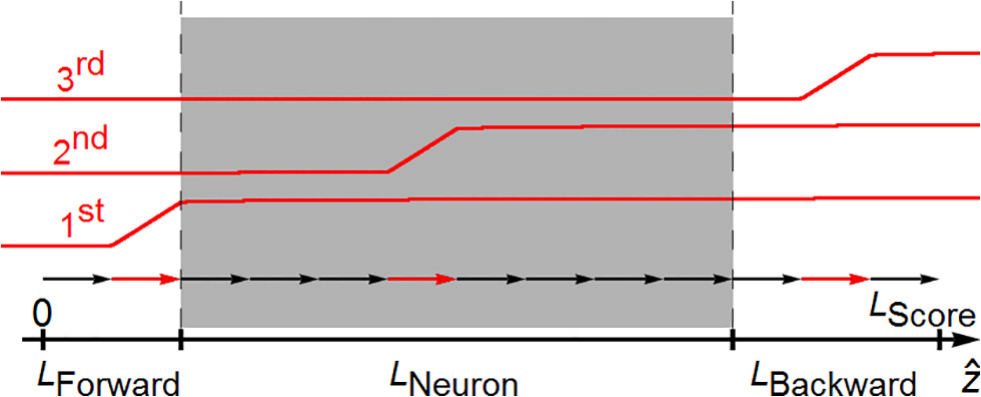
Longitudinal component of dose contribution to the inner, *Cyl*_*Neuron*_ cylinder section. Along the *z*-axis, the gray shaded rectangle represents the length of the *Cyl*_*Neuron*_ and each sequential arrow (black and red) is made up of random histories of length *L*_*Track*_ (20 μm) to establish a particle beam in *Cyl*_*Score*_. Axis length of *Cyl*_*Score*_, *L*_*Score*_ is the sum of *L*_*Forward*_, *L*_*Neuron*_ and *L*_*Backward*_. Three red arrows are chosen to show contribution of these tracks to the ED in *Cyl*_*Neuron*_. Corresponding three red curves are the longitudinal component of translated dose distribution of normalized deposited energies. The longitudinal contribution of any history (red curve) to the *Cyl*_*Neuron*_ (shaded area) is the absolute value of the differences of these curves at entrance and exit points to the *Cyl*_*Neuron*_ (dashed lines). The contributions of all the tracks (13 in this figure) are added and the sum is divided by the number of tracks bounded by the length *L*_*Neuron*_ (8 in the figure). The tracks along both *L*_*Forward*_ and *L*_*Backward*_ act as the longitudinal component of build-up materials to reach electronic equilibrium in *L*_*Neuron*_.

The entrance point of the particle beam is bounded by the base surface of *Cyl*_*Score*_. A random entrance point (*x*_*Rand*,*i*_, *y*_*Rand*,*i*_, 0) is chosen at each trial *i*. The construction of an ionizing beam depends on the *z*-axis path length, *L*_*Score*,*i*_, as total number of (*N*_*History*,*i*_) different histories with length, *L*_*track*_, are read randomly from the particle track library created by the RITRACKS software. The total number of histories for each trial is found by *N*_*History*,*i*_ = *ceiling*(*L*_*Score*,*i*_/*L*_*track*_). The random histories are translated to (*x*_*Rand*,*i*_, *y*_*Rand*,*i*_, *k×L*_*track*_) point for histories spanning *k* = 0,1,…,*N*_*History*,*i*_ − 1 to create a beam of length *N*_*History*,*i*_ × *L*_*track*_. A finite length simulated beam is composed of linearly added track beams with *L*_*track*_ that goes through the cylinder volume perpendicular to the surface. A discrete number of beams, *N*_*History*_, to cover the total axis length *L*_*Score*_ and corresponding distances, *L*_*Forward*_, *L*_*Backward*_ and *L*_*Neuron*_ were chosen with values for *L*_*Forward*_ and *L*_*Backward*_ given different values for each particle and are given in Table A of S1 Text. **Fig 3** illustrates the methods for constructing each trial from the library generated using RITRACK. As discussed above the longitudinal range, *L*_*Neuron*,*i*_ was variable at each trial and *L*_*Neuron*_ = 140 μm is taken as the range to ensure a constant value in this calculation between the minimum thickness of the neuron 35 μm and maximum diagonal distance 256 μm. The start point of a track is plotted as the *z* = 0 point on the LCDF plot in **Fig 3**. Contribution of each track in a beam to the gray shaded longitudinal volume is calculated by how much of LCDF(*L*_*k*_) contributes to the volume of interest where *L*_*i*_ are the distances of zero point of the track to the entrance (*L*_*1*_) and exit point (*L*_*2*_) distance of the volume of interest (dashed lines in **Fig 3**).

In a different manner than that of HZE particle beam where the tracks are added subsequently, electrons are described by keeping the same random entry point *i* (*x*_*Rand*,*i*_, *y*_*Rand*,*i*_, *z*), propagating along the +*z*-axis, while the *z*-coordinate of the next random track starts the *z*-coordinate of the last voxel of the previous track. In this way a continuation of the electron along the +*z*-axis is provided, as the first track is always entering *L*_*Forward*_ upstream and the last track leaving the *Cyl*_*Score*_ at *L*_*Backward*_ from the neuron at each trial. The mean last voxel point on *z*-axis is 46.4 μm at the library. The total energy lost along the initial propagation direction (*z*-axis) is 10 keV that gives and average LET value of 0.215 keV/μm for electrons.

A coincidence algorithm between the voxels of each beam and the neuronal segments is made by testing if the voxel coordinates are inside any of the cylindrical segments including soma, dendrites, spines and filopodia. If there is any coincidence events these coordinates are back rotated and translated to initial and fixed neuronal coordinate configuration and recorded for that trial. A library is created over many trials and results are summarized in **Table 2**. The “fluence dose” is calculated over all the trials as *Fluence Dose* = *Number of trials* × *Dose per beam*. The coincidence results library is further sampled to quantify the dose to dendrites, spines and filopodia.

**Table 2:** A summary of simulation results to create the coincidence libraries.

Charged Particle	Number of trials	Fluence dose (Gy)	Neuron dose (Gy)	LET of the histories	Dose per beam (Gy)
^56^Fe (600 MeV/u)	20,000	2.27	1.79	172.4 keV/μm	1.14×10^−4^
^12^C (300 MeV/u)	405,000	2.64	2.31	12.9 keV/μm	6.51×10^−6^
^1^H (250 MeV)	2.1×10^6^	1.35	1.16	398.2 eV/μm	6.40×10^−7^
e^-^ (500 to 490 keV)	12×10^6^	1.30	0.92	215.3 eV/μm	1.08×10^−7^

*Dose per beam* = 0.16×LET(keV/μm)/*A*_*Score*_(μm^2^) where *A*_*Score*_ $=\pi R_{Score}^{2}$ with *R*_*Score*_ = (278, 318, 178, 318) μm for (^56^Fe, ^12^C, ^1^H, e^-^) particles.

The probability of coincidence per beam over a trial between any voxel of a beam and the test neuron is low; (14.92, 1.937, 1.66, 0.4871)% for (^56^Fe, ^12^C, ^1^H, e^-^) beams because the neuron fills only a small fraction of the fluence volume defined by *Cyl*_*Score*_ and the area of the fluence cylinder ($A_{Score}=\pi R_{Score}^{2}$) is kept large enough to have electronic equilibrium for the embedded neuron bounded by *Cyl*_*Neuron*_. The computational load of the coincidence of events per beam and neuronal segments at each trial can be estimated by giving the average number of voxels (36137, 4517, 234, 292) per 20 μm (*L*_*track*_) for (^56^Fe, ^12^C, ^1^H, e^-^) particles and the number of histories, *N*_*History*,*i*_ per beam for an average *L*_*Score*_ of 300 μm which is found as 15 (7 for electrons; mean(*L*_*Track*_)~46.4 μm). The number of neuronal segments in this example is 2610. The percentage of success defined as if one or more voxels of a beam is bounded by neuronal segment boundaries in a trial is given above.

### Sampling of energy deposition event over trials for given dose

The particle fluence to *Cyl*_*Score*_ is calculated as the ratio of the number of trials to the cylinder base area *A*_*Score*_ the beam impinges, and the neuron dose is calculated by summing all the voxel ED events where voxels coordinates coincide with neuronal segments in the trials. The dose in Gray of a single beam is determined by 0.16 × *LET* (*keV*/*μm*)/*A*_*Score*_(*μm* ^2^) where LET is calculated over the average value of all the ED events of histories generated by RITRACKS and used in the simulations in **Table 2**.

Statistical realizations of any dose can be sampled from the trial library; first by finding the fluence, hence the mean number of beams (< *N*_*Dose*_ >), then drawing a random number for that beam (*N*_*Dose*,*P*_) from a Poisson distribution to reflect variability at low fluence. Another set of distinct *N*_*Dose*,*P*_ number of random integers can be drawn from the number of trials library for that beam and if there are any registered coincidence files; hits, the coordinates and energy values of the voxels can be placed in neuronal segments. These results can be displayed or further evaluated, such as finding the dose in the neuronal segments or counting how many voxels including coordinates and energies are located in dendrites and spines or filopodia.

### Considerations on electronic equilibrium

The δ-rays from HZE particles can extend for many millimeters from the particles track (**Fig 2**), both radially from the surface of entrance and longitudinally in backward and forward directions. The larger volume *Cyl*_*Score*_, containing the volume of interest at which test neuron and ED events are recorded, is utilized to reach electronic equilibrium in *Cyl*_*Neuron*_. Extending radial and longitudinal size of the *Cyl*_*Score*_ volume lengthens the time of simulations, and the size of electronic equilibrium.

The test neuron dose calculated from the fluence to the surface of *Cyl*_*Score*_ and measured by adding all the ED events bounded by the neuron over all the trials are quantified by a predicted value of the *Cyl*_*Neuron*_ volume by electronic equilibrium approach. The details of the numerical study are given in the S1 Text. Briefly, orthogonal radial and longitudinal normalized deposited energy functions in **Fig 2** are numerically calculated for each HZE particle and electrons considered in this study. The spatial extent of each particle beam entering the *Cyl*_*Score*_ cylinder surface and propagating along its z-axis is treated as unit sources and their radial and longitudinal components bounded by the *Cyl*_*Neuron*_ numerically calculated to predict the measured dose.

## Results

### Quantification of voxel energy distribution

**Fig 4:**
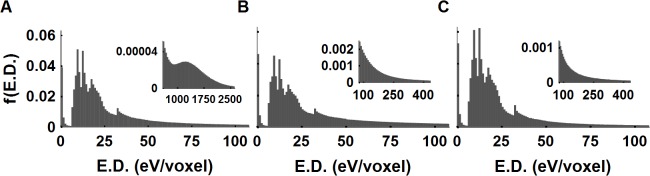
Distribution of energy deposition, f(ED) deposited in 20 nm^3^ voxels by HZE particles and protons. The ED distribution of the 20 nm^3^ voxel for (^56^Fe, ^12^C, ^1^H) particles (A-C) are similar at lower ED per voxel (e.g., <60 eV). The high ED distribution tails, for example, the highest 5% of the voxel ED carry (56.0, 31.2, 36.8 35.9)% of the total deposited voxel energies. The LET values of track libraries are (172.4, 12.9, 0.4) keV/μm. High LET ^56^Fe particles have a very high voxel ED (a hump in the inset) that is located along the primary track while other charged particles voxel EDs decrease monotonically for ED>30 eV.

The ED distribution within 20 nm^3^ voxel volumes generated by the RITRACKS code [29,30] for the ^56^Fe, ^12^C and ^1^H particle at (600 MeV/u, 300 MeV/u, 250 MeV) are plotted in **Fig 4**, with the mean energy deposited of the particles found as (95.5, 57.3, 34.0) eV, respectively. The peak positions of the histograms and amplitudes occur for ED lower than, e.g. 60 eV/voxel in **Fig 4A–4D**. This corresponds to the discreteness of the number of ED events occurring in the 20 nm^3^ voxels [31]. ^56^Fe particles were found to have a very high voxel ED tail (>1000 eV/voxel) (**Fig 4A**). The number of voxels with over 1000 eV/voxel accounts for only 2.6% of the total voxels, but contains 44% of the total ED. These large ED events are located within 100 nm lateral to the ^56^Fe particle track. ^12^C and ^1^H particles were not found to have similar high ED tails, but instead a monotonic decrease with increasing ED occurs. For ^12^C and ^1^H particles, the highest 2% of voxel ED are larger than (470, 229) eV/voxel and account for the (17.3, 22.8)% of the total deposited energies, respectively. Predictions of ED distributions for 0.5 MeV electrons (result not shown) where found to be very similar to the 250 MeV protons.

**Fig 5:**
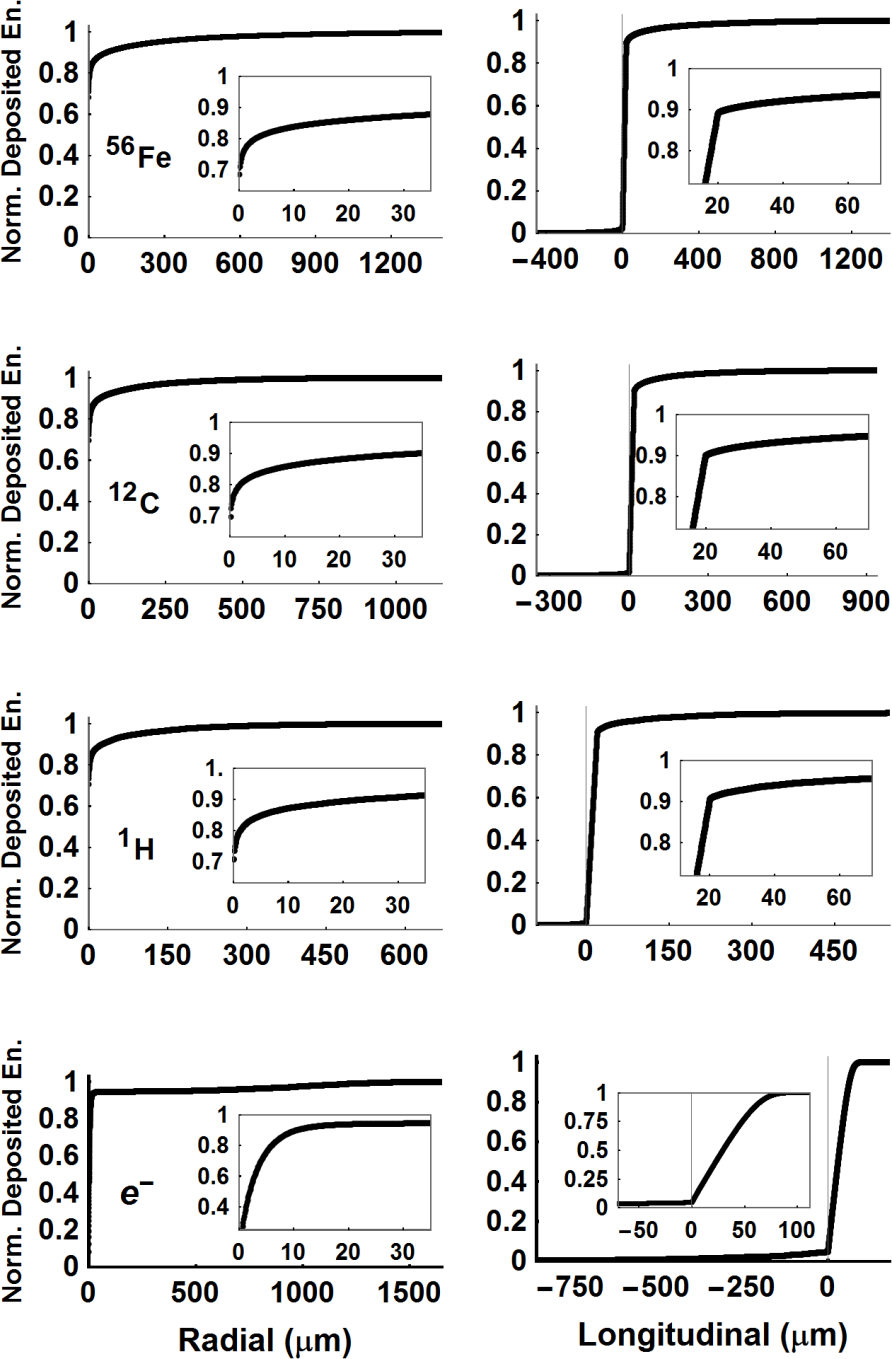
Normalized radial and longitudinal deposited energy plots. Three-dimensional dose distribution from a particle beam is mapped to two orthogonal functions; longitudinal and radial dose distributions from the source. A kernel representing the normalized cumulative dose deposition events of 20 μm histories, generated by RITRACKS are calculated from the irradiation library, detailed in the S1 Text. Each point on the beam is propagating along *Cyl*_*Score*_ axis treated as a source and their contribution to the volume of interest, *Cyl*_*Neuron*_ is calculated. The kernels of the orthogonal directions are presented on the first column for the radial and the second column for the longitudinal component for the particles. A large portion of the energy deposition for HZE particles occurs within 100 nm radially along the propagation direction (68.3, 69.7, 70.8)% for (^56^Fe, ^12^C, ^1^H) particles at (600 MeV/u, 300 MeV/u, 250 MeV). The radial and longitudinal extend arise from the δ-rays whose range increases with particle kinetic energy. Electrons that start their motion on *z*-axis at 500 keV are strongly scattered radially and backward directions as their energy reaches 490 keV in the fourth row. Insets in the figures illuminate difficulty of reaching full electronic equilibrium by expanding build-up region (*R*_*Gap*_, *L*_*Forward*_, *L*_*Backward*_) beyond initial rapid rise (e.g. 30 μm).

Unlike ^56^Fe particles, which have the mean voxel ED 100 nm lateral to particle track of 190.7 eV/voxel, ^12^C particles show a moderate increase (62.7 eV/voxel) while a slight decrease was found for ^1^H (30.2 eV/voxel). However, the number of voxels within 100 nm accounts for (34.1, 63.6, 79.6)% of total voxels for (^56^Fe, ^12^C, ^1^H) particles, respectively. These overall voxel distributions give rise to large ED distribution within 100 nm vicinity of the particle tracks as presented in radial distribution of spatial ED profiles in **Fig 5**. In considering electrons the simulation initially starts at the (0,0,0) coordinate, and propagates along the +*z*-direction with the initial energy of 500 keV as detailed in Materials and Methods section. The mean ED per 20 nm voxels between 500 to 490 keV is found as 34.09 eV/voxel. The high energy tail of the voxel ED larger than 222 eV/voxel accounts for 2% of the voxels and 22.6% of the total deposited energy in **Fig 5D**. In comparison, the 20 nm^3^ voxel ED distribution for the ^1^H particles and electrons at these energies are very similar.

### Geometrical considerations: microscopic dose deposition in neurons

Results show that modifying the voxel size changes the ED distribution as differences in stochastic coulomb interactions occur in the voxel as the volume is changed. However, normalized radial and longitudinal energy deposition distributions in **Fig 5** were not found to change with voxel size. The neuronal segment and compartment volumes, the smallest being a spine segment of 125 nm^3^ and spine volume of 355 nm^3^ in this study, are much larger than the 20 nm^3^ voxels. Absorbed dose to the test neuron or any compartments including soma, dendrites, spines or filopodia can therefore be found by normalizing the total energy of voxel EDs located within the compartment used in the numerical simulation, assuming the cellular density is the same as water.

The probability of success defined as the occurrence of an ED event in the test neuron that is hit by a beam is (14.92, 1.94, 1.66, 0.49)% for (^56^Fe, ^12^C, ^1^H, e^-^), and this ratio depends on the number of voxels per track, hence LET and the extent of the geometrical distances between *Cyl*_*Neuron*_ and *Cyl*_*Score*_ to reach electronic equilibrium. A better electronic equilibrium would be reached by allowing for larger dimensions of build-up distances, however would result in a significant increase in simulation time and a decrease in the probability of hit per beam. The average number of voxels that coincide with the test neuron, if there is any hit, is (101.4, 89.2, 17.2, 8.2) for (^56^Fe, ^12^C, ^1^H, e^-^) particles, respectively. The mean ED to the voxels that are in coincidence with the test neuron are (104.57, 58.11, 34.19, 34.01) eV/voxel for (^56^Fe, ^12^C, ^1^H, e^-^) particles. The mean ED of voxels is larger lateral to the ^56^Fe and ^12^C particle tracks and main particle tracks are well confined by the simulation geometry but δ-ray events that extend over hundreds of microns in **Fig 5** may not be bound by the volumes taken in this study. This also shows the difficulty of achieving electronic equilibrium for high LET particles in small test volumes. Normalization of sampled total voxel energy deposition by the number of voxels within the neuron increases the mean ED for ^56^Fe and ^12^C particles.

### Neuronal microdosimetry of different LET charged particles

**Fig 6:**
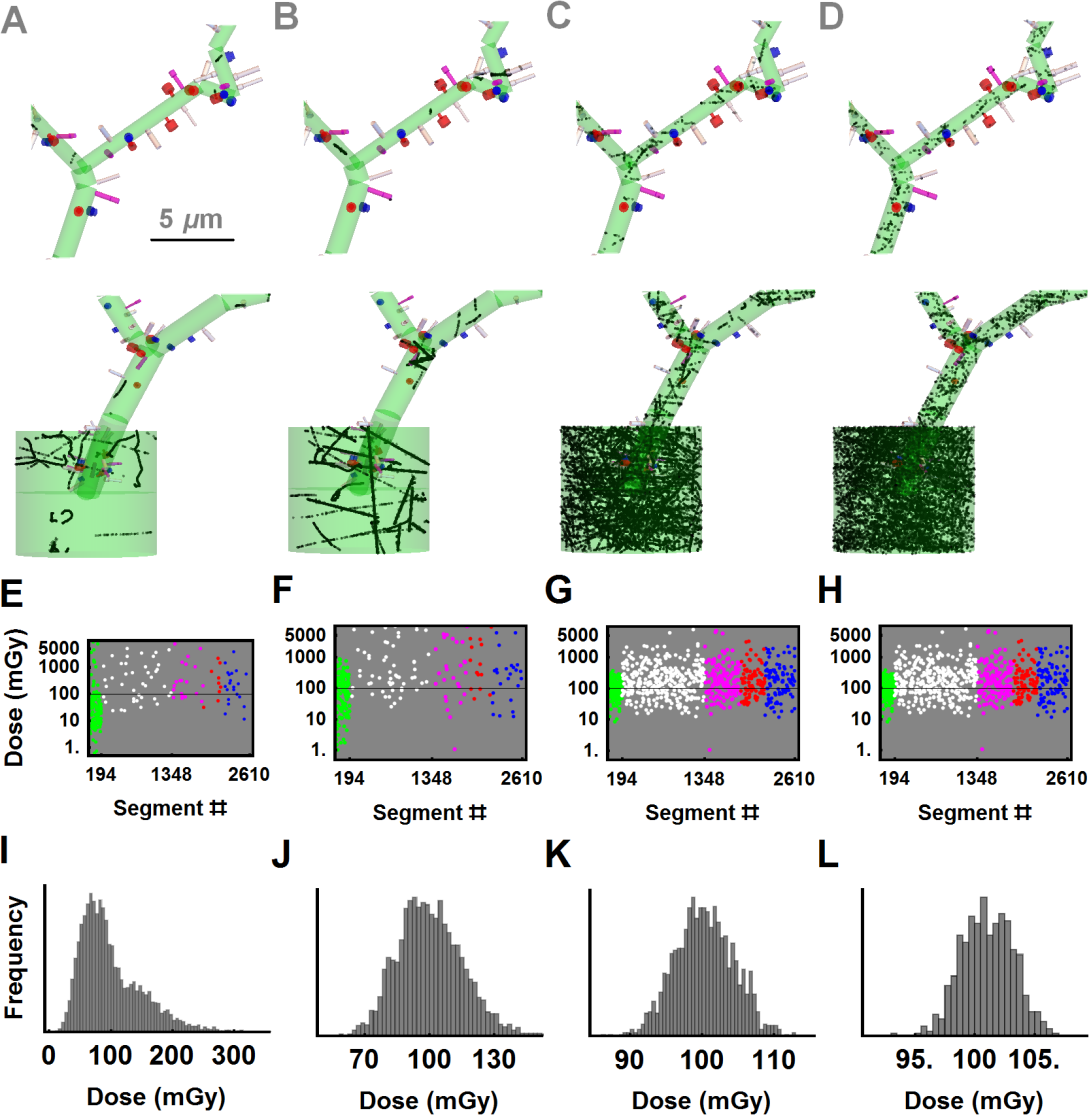
A sample profile of dose deposition at 100 mGy. A statistical realization of voxel dose distribution for 100 mGy neuronal dose plotted in panels (A) through (D) for iron, carbon, proton and electron beams at (600 MeV/u, 300 MeV/u, 250 MeV, 500–450 keV) respectively. Segment dose distribution in (E-H) is highlighted with the colors for dendrites (green), filopodia (white), long (magenta), mushroom (red) and stubby (blue) spines. These segment numbers on the test neuron are {(3–194), (195–1348), (1349–1848), (1849–2184), (2185–2610)} for dendrites, filopodia, and long, mushroom, stubby spines. First and second segments make up the soma. Panels (I) to (L) show predictions of the frequency of deposited dose for iron, carbon, proton and electron beams corresponding to Panels (A) to D), respectively.

A statistical realization of ED to the test neuron at given dose is sampled from the coincidence library and the pooled results are displayed in **Fig 6**. A constant fluence value, as the number of particles for a given neuronal dose, is calculated and the random simulations that result from Poisson statistics are selected from the coincidence library as a statistical realization (Materials and Methods). An example of such a sampling to dendritic branches, spines, filopodia and soma for 100 mGy neuronal dose is shown in **Fig 6** for (^56^Fe, ^12^C, ^1^H, e^-^) particle beams. The neuronal dose of the realization is (102, 108, 106, 100) mGy in **Fig 6** panels, A-D. If there is any coincidence of ED to any neuronal segments then the segmental dose is also calculated for the same sample in **Fig 6**E and **6**F for the (^56^Fe, ^12^C, ^1^H, e^-^) particles. The color code of the segment dose distribution represents dendrites (green), filopodia (white), long (magenta), mushroom (red) and stubby (blue) spines. The total neuronal dose shows variability for the same fluence between each statistical sampling. Frequency histograms for four charged particles at 100 mGy are shown in **Fig 6**I–**6**L.

A close inspection of the dose distribution in **Fig 6A** demonstrates that it results predominantly from δ-rays, and not the primary particle track. There are multiples of primary ^12^C tracks depicted as voxelated, dense straight lines both in the soma and the main dendritic branch in **Fig 6B**. There are many primary ^1^H beams traversing the soma, dendrites and some of the spines and filopodia in **Fig 6C**. Likewise, electron ED events in **Fig 6D** nearly fill every segment randomly, including the spines and filopodia. The number of neuronal segments hit and variability of segmental dose are correlated with the LET of the charged particles. The number of dendritic segments (green dots) out of 192 segments that received any hit are (126, 140, 191, 192) for (^56^Fe, ^12^C, ^1^H, e^-^) beams in **Fig 6E–6H**. Dendritic segmental doses show great variability for high LET ^56^Fe and ^12^C particles and there are segments receiving over 1 Gy doses for the ^56^Fe beam. Spines and filopodia with submicron volumes receive fewer hits for high LET particles compared to electrons or protons. However, average segment doses for these compartments are higher for high LET particles. A systemic statistical evaluation of these observations is given in the next section. The variability, quantified as standard deviation (SD) in total neuronal dose is (49.5, 14.8, 4.0, 1.7) mGy in **Fig 6I–6L**.

### Coefficient of variation of neuronal dose decreases with dose

**Fig 7:**
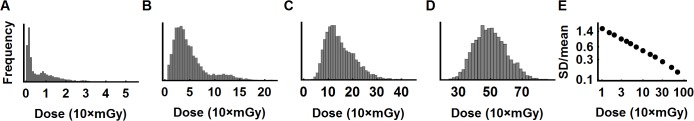
The ratio of standard deviation to mean dose (SD/mean) decreases with neuronal dose. Variability in neuronal dose between sampling trials for the same fluence dose (10, 50, 150, 500) mGy in panel (A) through (D) for ^56^Fe at 600 (MeV/u) are shown. The mean and SD values are (10.2, 50.5, 149.8, 499.5) mGy, (16.7, 36.3, 59.8, 98.0) mGy, respectively. The coefficient of variation (CV = SD/mean) is calculated as (1.64, 0.72, 0.40, 0.20) at these doses. As the statistical sampling is extended to other neuronal doses in panel (E) a power low relation indicates a straight line on a log-log scale plot.

A useful measure to quantify variability of neuronal dose at given fluence is to introduce a coefficient of variation (CV) as the ratio of standard deviation to mean (SD/mean) neuronal dose over many sampling trials. In this way, the increase in standard deviation with increasing dose is represented as a unitless quantity (CV) and the coefficient of variation can be analyzed for the same neuron between different LET particles. As it is only displayed for the highest LET particle, ^56^Fe in **Fig 7** for different neuronal doses the SD increases with dose but the ratio of SD to mean decreases.

Similar results as found for Fe particles are also obtained for the other three ionizing particles used in this paper. A power law relation can be fit to the form of *SD/mean* = *amplitude* × *Dose* ^*power*^ where *Dose*, *amplitude* and *power* are the mean dose and the amplitude and exponent of the power law relation. The values for *amplitude* (55.50, 15.74, 4.55, 3.10) and *power* (˗0.5266, ˗0.5138, ˗0.5271, ˗0.5345) are fitted for (^56^Fe, ^12^C, ^1^H, e^-^) particles, respectively. The amplitude term (*amplitude*) signifies the variability of SD at the same mean dose between different LET particles as in **Fig 6I–6L** and there is a qualitative relation between LET and amplitude terms; high LET particles have higher amplitude and low LET particles have lower amplitude. Details of this relationship likely depend on the neuron structure and volume. On the other hand, the decrease in the power law relation fits to the same exponent (˗0.526±0.0091) for all particles considered. The numerical value of the exponent depends on the volume of the neuron, its compartments, LET of the particles and fluence dose that will be investigated in the next section.

These results show that for ^56^Fe particles at low dose, e.g. 10 mGy with large CV of 1.64 in **Fig 7A**, 35% of the instances the neuronal dose is smaller than 2 mGy. On the other hand, the mean dose is larger than 50 mGy at 2.8% of the trials and even larger than 100 mGy at 0.84% of the sampled trials. The frequency of these neuronal doses can be understood by frequency of HZE particle tracks passing through the soma. For example, an ^56^Fe particle track with LET of 172 keV/μm crossing a path length of 10 μm of the soma and depositing 80% of its energy to the neuron would result in 81.5 mGy neuronal dose for a neuron with volume of 2700 μm^3^. A similar calculation for two particle tracks crossing the soma but shorter cellular path lengths could give similar results. This example indicates the larger variability that will occur for low dose exposures of neuronal structures, especially for HZE particles and other high LET radiation.

### Compartmental coincidence statistics

The neuron is represented as cylindrical segments and voxel ED events are recorded in all the segments. Functional compartments including soma, spines and filopodia are represented as two interconnected cylinders. Defining two corresponding segments as a unit compartment (soma, spine, filopodium) helps to characterize experimentally relevant observations including the number of spines, filopodia pruned or neurons survived following irradiation of biological samples [13,19,23,24]. Joining two sub-micron segment volumes to represent a spine and a filopodium compartment also increases total compartment volume hence reduces the standard deviation. Each dendritic segment is interpreted as a compartment in this study. The sampling statistics represents dose value as mean±SD at given fluence dose over many trials in soma, dendrites, spines and filopodia for (^56^Fe, ^12^C, ^1^H, e^-^) particles and the ratio of dendrites, spines and filopodia are hit presented in **Table 3**. The dose at each compartment is found by normalizing the total ED energies to the corresponding compartment volumes. The percentage of hits is reported as the number of compartments hit by ED events to total number of compartments of relevant type. The soma with a large volume (1129.6 μm^3^) is always hit at the doses considered in **Table 3**. The statistics of other compartments of the test neuron is as follows. The volumes (mean±SD) of (dendrite, spine, filopodium) compartments are (8.12±5.91, 0.119±0.039, 0.114±0.028) μm^3^. The total number of (dendrite, spine, filopodium) compartments are (192, 631, 577) giving rise to total volumes of (1558.9, 75.1, 65.8) μm^3^, respectively.

**Table 3:** Coincidence statistics of selected fluence doses in the test neuron compartments.

Fluence dose (mGy)	Neuron dose (mGy)	Compartmental dose of particle hits: mean±SD (mGy)				% Volumes hit: mean±SD		
		Soma	Dendrites	Spines	Filopodia	Dendrites	Spines	Filopodia
**Iron**								
10	11.3±18.1	9.4±38.2	110.5±150.6	2105.3±6549.1	1660.6±4755.1	10.95±3.28	0.59±0.35	0.61±0.37
50	53.6±44.0	45.3±89.4	145.7±94.3	1940.2±2534.5	1586.5±2089.4	42.29±4.82	2.97±0.78	3.07±0.80
100	99.5±56.1	81.2±120.2	186.6±87.6	2006.5±1646.2	1805.5±1661.3	64.13±4.12	5.71±1.03	5.82±1.10
150	151.2±64.6	110.9±127.4	251.6±90.0	2006.6±1349.7	1781.5±1365.9	77.05±3.12	8.40±1.11	8.70±1.28
200	207.8±77.1	172.0±154.0	298.5±97.0	2193.3±1270.9	1879.5±1169.2	84.04±2.82	11.31±1.36	11.49±1.45
400	412.7±105.6	331.8±197.4	517.3±116.1	2158.4±822.9	1904.1±753.1	96.42±1.37	21.26±1.67	21.33±1.69
**Carbon**								
10	10.5±5.1	9.0±10.3	73.1±26.2	1178.8±1038.4	1347.3±1136.9	15.63±2.85	0.79±0.36	0.97±0.42
50	51.7±11.7	44.2±22.0	104.0±17.6	1265.4±422.6	1303.5±415.0	53.45±3.62	3.91±0.75	4.65±0.89
100	104.2±18.3	88.2±35.8	151.3±18.7	1354.4±315.7	1340.9±320.5	75.16±3.17	7.69±1.03	9.10±1.41
150	157.1±21.3	137.0±40.7	196.0±20.7	1366.7±281.2	1376.1±251.8	85.57±2.54	11.26±1.35	13.10±1.40
200	209.5±23.2	181.6±46.5	247.2±22.3	1390.2±235.1	1389.0±221.9	91.21±1.88	14.75±1.45	17.43±1.57
400	415.5±30.5	356.7±60.4	458.1±28.6	1519.7±170.3	1547.1±175.0	97.99±0.93	26.98±1.72	31.52±1.98
**Proton**								
10	10.4±1.4	9.1±2.7	16.3±2.3	180.0±62.8	189.2±78.8	68.73±3.33	5.84±1.00	6.37±1.03
50	52.3±3.7	45.5±6.6	58.4±4.1	201.0±31.5	212.8±34.0	98.24±0.90	25.67±1.74	27.97±1.84
100	104.0±4.3	89.6±8.3	114.7±5.2	231.6±24.3	254.2±32.0	99.80±0.32	44.72±1.97	48.11±2.06
150	156.1±5.4	135.0±9.4	171.5±6.0	267.2±21.8	293.8±28.3	99.98±0.12	58.56±2.07	62.29±1.75
200	207.9±6.1	179.6±10.8	228.8±7.1	300.0±21.8	333.5±28.4	100.0±0.0	69.16±1.60	72.61±1.85
400	416.2±8.5	359.6±15.7	458.3±9.2	459.6±20.6	527.1±28.0	100.0±0.0	89.76±1.00	91.86±1.09
**Electron**								
10	10.2±1.0	8.7±1.7	12.9±1.4	115.6±40.2	111.9±36.2	88.30±2.14	9.98±1.16	10.30±1.32
50	51.7±2.1	44.1±3.8	57.5±3.0	142.3±20.7	139.3±18.9	99.82±0.30	40.16±1.95	41.71±2.12
100	103.4±2.9	87.8±5.5	115.3±3.9	181.6±19.3	176.6±16.9	99.99±0.08	63.69±1.79	65.21±1.95
150	154.8±3.2	131.9±6.1	172.7±5.1	221.0±17.4	218.3±18.2	99.99±0.03	77.29±1.63	78.99±1.55
200	206.6±3.4	176.1±6.5	230.2±4.7	266.7±17.5	263.4±18.5	100.0±0.0	85.75±1.42	87.09±1.46
400	413.2±4.5	352.0±7.4	459.0±6.0	471.5±17.2	473.7±17.4	100.0±0.0	97.35±0.62	97.83±0.47

In general, the percentage of a compartment type that is hit and the mean compartment volume are inversely proportional for all the particles in **Table 3**, and reaches unity at higher neuronal doses starting with the lower LET particles; electrons and protons. The probability of a hit of any compartment type is expected to be the ratio of the compartment type volume to the total neuron volume if voxel distribution is uniform. This inverse relation has the lowest ratio for the highest LET particle; ^56^Fe. In addition, the mean dose of any compartment type hit is inversely proportional to the mean compartment volume and the mean compartment dose is highest for the highest LET particle. Taken together, this relation leads to the same mean neuronal dose for all the particles. An interesting observation is the nearly constant and high mean compartmental dose at 1 to 400 mGy in spines and filopodia for ^56^Fe and ^12^C particles. The variability in mean dose between trials decreases in these sub-micron volumes with increasing fluence dose. The low LET protons and electrons achieve homogenous voxel dose distributions, however in comparison for high LET iron and carbon particles the ratio of spine, filopodia dose to neuron dose is still an order of magnitude higher at 10 mGy and only approaches unity at the higher doses in **Table 3**.

The variability in compartmental dose between types of compartments, particles of different LET values over a range of fluence dose require further analysis. The overall neuronal dose is distributed with large variability and the results in **Table 3** support that the highly sampled data in **Fig 7**; the coefficient of variation of neuronal dose decreases with dose, close to an inverse square root relation. The sampled data in **Table 3** support an exact *power* value of ˗0.5 for the neuron (˗0.5045±0.0404) and the soma (˗0.5084±0.0158) for all four particles considered. The dendrites with much smaller mean volumes have the *power* value of (˗1.692, ˗1.269, ˗0.5769, ˗0.5352) for the (^56^Fe, ^12^C, ^1^H, e^-^) particles. This indicates that lower LET particles approach the ˗0.5 exponent value in small dendritic segments. The power law relation for spines and filopodia with sub-micron volumes are fitted to *power* value of ˗2.658±1.281 and ˗2.579±0.673 for all the particles. All the amplitude terms on these fits are ordered from high to low as the LET values of the four particles, which is in agreement with **Fig 7**.

## Discussion

### A computational method for microdosimetry of *in silico* neuronal structures is developed

The *in silico* model developed unifies stochastic energy deposition events of different radiation qualities and doses, and morphological data of neurons to explore radiation damage to neurons and their sub-compartments. The Monte-Carlo track structure simulation code, RITRACKs, characterizes the stochastics of energy deposition events of given charged particle at predetermined voxel volumes. Each Monte-Carlo simulation of a 20 micron track segment generated for the radiation library in this work represents a statistical sample for the subsequent calculations. The accuracy of the RITRACKs has been described in terms of comparisons of average quantities that result from statistical samples such as LET and radial dose distributions and for the case of a ED in fixed spherical or right cylindrical target volumes in previous work [29,30], and in tests of similar quantities in analysis of electronic equilibrium described in this work. The neuron morphologies for hippocampal granule cells in C57BL6/Trk.T1 deficient mice [32,33] represent deterministic samples, with the exception of the number and positions of spines and filopodia along the dendrites as well as the orientation of a neuron cell relative to the particle track. Coincidence events of ED in voxels and neuronal segments are simulated to determine dose at given neuronal compartments. In this approach, layers of randomization steps are applied to statistically capture possible outcomes. Stochastics of arrival of charged particles, microscopic ED events of a particle, relative orientation of a particle track and the neuron and sampling of possible outcomes at given fluence dose are studied for a test neuron. The procedure with higher computational power can be extended to *in silico* brain structures or real anatomical morphological data, such as sliced and confocal scanned control and irradiated brain tissues that can be computationally processed and ED profiles at given fluence dose can be compared with irradiated structures to infer changes in neuronal anatomy for a particular type of particle and its kinetic energy.

Statistical correlates of these predictions can be quantified by the ED distribution and dose in microscopic compartments including spines, filopodia, dendrites and soma. A useful outcome of this computational study would be to associate structural damage quantified as changes in the number of spines, filopodia, total dendritic length, total number of branches and cell number on control versus irradiated tissue samples of the same anatomical region of experimental animals. These changes can be interpreted as normal tissue effects in neurons for different types of ionizing radiation, at a given particle fluence or dose.

It is widely known that changes in neuronal and spine structure as well as changes in the expression of various neurotransmitters account for the cognitive decline observed in normal aging and in the early stages of numerous neurodegenerative diseases such as Alzheimer’s disease and Parkinson’s disease [34–37]. Many of the consequences of the foregoing insults to the brain can be linked to alterations in the outgrowth and elongation of dendrites, branching of the dendrites, number of dendritic endings and cell body area [34–37]. These morphometric changes in neurons depend on a variety of signaling cascades that regulate the amount of remodeling and neurite outgrowth in response to specific stimuli [38–40]. Neuronal cell morphology is modulated by a variety of intrinsic and extrinsic stimuli such as trophic factors, electrical activity, synaptogenesis, dendritic arborization, functional maturation and differentiation of neurons [40]. Morphologic remodeling of dendrites usually reflects a response to adverse conditions that trigger adaptive remodeling within the compromised microenvironment of the brain [40]. In addition to changes in neuronal morphology, slight changes in dendritic spines can have tremendous effects on synaptic function and the connectivity within neuronal circuits [34–37]. Dendritic spines are small membranous protrusions on neuronal dendrites that receive synaptic input from axon terminals and represent the structural correlates of learning and memory [37–43]. Dendritic spinogenesis and remodeling underscores the structural and synaptic plasticity critical for CNS function, as spine enlargement is tied to long-term potentiation, while spine shrinkage corresponds to long-term depression [38] Notably, disruptions in dendritic spine morphology, including the shape, size and number of spines, have been found in several developmental and neurodegenerative brain disorders [37–43] suggesting that dendritic spines are likely to serve as key players in the pathogenesis of radiation-induced cognitive dysfunction.

### Compartmental dose as a determinant of cellular injury

The tree-like structure of neurons requires morphological constraints to be included in analysis of the response of neurons to ionizing radiation. In general, dendritic branches extend over a hundred microns from soma. The protruding spines from dendrites establish diffusion limited compartments for ions and cellular proteins [44,45], while dynamic filopodia search for new connections sites to establish synapses [46]. The soma is spherical in shape and contains most of the organelles surrounding the nucleus. Organelles that are synthesized at the nucleus are transported to dendritic branches including spines to support and maintain cellular functions [47]. A direct target at the soma for microscopic ED events can be the nuclear DNA. The main dendritic branches extending from the soma support distal dendritic branches, spines and filopodia. Cellular damage to dendritic architecture by ionizing radiation could impact directly or indirectly the transport of cellular organelles to distal points [48], local protein synthesis, and polymerization of actin based cytoskeleton to maintain spines and filopodia [49]. Cellular injury on main dendritic branches, proximal to soma as in **Fig 6A–6D**, may initiate cell death pathways and cause collapse of the whole neuron [50].

Differences in radiation sensitivity and morphology of the three types of spines [13,19] indicate dynamic molecular processes between cellular injury and spine pruning within micron scale compartments. Dendrite and spine pruning can change the electrophysiological properties of neurons, for example post synaptic input currents at spines established by spine properties change with spine numbers at excitatory synapses. These membrane currents coded as a change in membrane potential conducted along the dendrites to soma would change action potential kinetics. Filopodia are the most ionizing radiation sensitive compartments and reduction in their numbers was found to increase with dose following proton and X-ray irradiation [13,19]. Because filopodia are the motile ends it would be interesting to investigate if there are any long term detrimental effects marked by molecular processes to generate new filopodia after irradiation [26].

### Interplay of LET of ionizing charged particles and neuronal morphology to determine neuronal cell kill and pruning

Energy deposition by charged particles is a stochastic process and as shown in this study (**Table 3**), compartments of a neuron can accumulate different amounts of energy for different particles types at given mean volume dose, **Fig 6I–6L**. The probability of hit of a compartment also varies greatly with LET for the same fluence dose. Voxel ED profiles of HZE particles and neuron morphology should be unified to have a conceptual picture of statistics presented in **Table** 3. HZE particles have large local ED distribution within a fraction of a micron range shown as radial increase in **Fig 5**. The fluence of HZE particles with large LET values, like ^56^Fe and ^12^C in this study, is sparse compared to low LET protons and electrons at the same dose as the number of particles aiming the anatomical structure is inversely scales with their LETs. Large mean voxel energies of high LET particles also contribute to heterogeneous dose distribution. In addition, the volume of a neuronal cell is compartmentalized and can extend great distances. Thus, a particle beam coinciding with different compartments of a neuron can have different structural damage patterns not adequately described by the particles absorbed dose or fluence.

The larger dose variability of a neuron, quantified as standard deviation at lower fluence for high LET particles in **Table 3**, indicates the number of primary particles crossing the soma. The low LET particles; protons, and electrons in this study establish a homogenous voxel distribution even at 1–100 mGy in **Table 3** and 100 mGy in **Fig 6**G and **6**H. Individual dendritic segments of a neuron with micron scale axial cross-section have a lower probability of hit than compared to the soma. The total deposited energy normalized by a dendritic segment volume (8.12±5.91 μm^3^ in this study) leads to an order or larger compartmental doses for dendrites.

High LET particles crossing the specific locations of dendritic branches may have different biological outcomes compared to low LET particles. Carbon has a LET value that is an order of magnitude smaller than iron and two orders of magnitude larger than protons or electrons. A comparative study between iron and carbon particles should include the variability between trials in **Table 3**. For example, for the fluence at 10 mGy, the mean values of probability of hit and dose in dendritic segments are almost the same for ^12^C and ^56^Fe particles (12.1, 11.4) mGy, respectively. The other parameters read from the **Table 3** are; the variability is represented as CV (1.36, 0.36), the mean dendritic dose (110, 73) mGy, and the mean number of dendritic segments hit (21, 30) for (^56^Fe, ^12^C) particles, respectively. The larger CV for ^56^Fe leads to a few dendritic segments to have an order higher segmental dose. The magnitude of CV for ^12^C particles would provide much uniform dendritic dose around the mean value. The outcomes of this statistics largely depend on neuronal response. Very high local compartmental dose may lead to irreparable cellular injury and pruning of dendritic branches. Determining dose and LET sensitivity of dendrite pruning thresholds for dendritic branches by experimental techniques and computational studies could help elucidate clinical implications in normal cell response under therapeutic radiation treatment and space radiation [20,21].

The smaller spine and filopodia compartments are hit more often by low LET protons and electrons than high LET particles at the same neuronal dose in **Table 3**. Sparse coincidence events of high LET particles with large compartmental doses may guarantee spine and filopodia pruning. However, details of spine pruning process and compartmental dose relation can shift the effectiveness of low LET particles at low doses. For example, roughly 50% of the spines and filopodia receive over 160 mGy dose at 100 mGy fluence dose by protons and electrons while the ratio is around 7% for carbon and iron over 1 Gy compartment dose in **Table** 3. A dose-response curve represented as a sigmoid function and mid-point value can determine the ratio of spines pruned and filopodia retracted by different LET particles and electrons at given fluence dose. Sensitivity of spine and filopodia pruning to low LET proton and electrons at 0.1 Gy [13] and at higher doses [16,19,23,26] motivates extensive experimental investigations complemented with computational microdosimetric studies as presented in this paper.

In conclusion, evidence for the sensitivity of spines to low and high LET radiation has now been reported [13,19,51], and suggests that radiation-induced reductions in spine density and morphologies are contributory if not causal for certain cognitive deficits found after cranial exposure. Even lower fluence of HZE particles [51] compared to photon or proton irradiation [13,19] were found to elicit impaired cognition coincident with dendritic alterations, suggesting that astronauts engaged in longer term space travel and exposed to charged particles are at heightened risk for developing adverse CNS deficits. The present study is the first investigation of the microscopic energy deposition that is induced by different radiation qualities across a wide range of dose or fluence in spines, dendrites and soma of hippocampal neurons. The results of the present study will be extremely valuable to support interpretation of experimental studies of radiation changes to cognition, and for developing detailed computational models of cognitive changes observed for different radiation qualities and doses.

## Supporting Information

S1 Fig Radial contribution of dose to inner, *Cyl*_*Neuron*_ volume (A) by particle fluence on *Cyl*_*Neuron*_ (B) and *Cy*_*lScore*_ (C) surface sections. (TIFF)

S1 Text Sensitivity study of charged particle electronic equilibrium for co-axial cylinders. (DOCX)
