# Supplementary figures and images for: Irradiation of Neurons with High-Energy Charged Particles: An In Silico Modeling Approach
Source: PLoS Comput Biol. 2015 Aug 7;11(8):e1004428. doi: 10.1371/journal.pcbi.1004428 (PMC4529238; doi:10.1371/journal.pcbi.1004428)

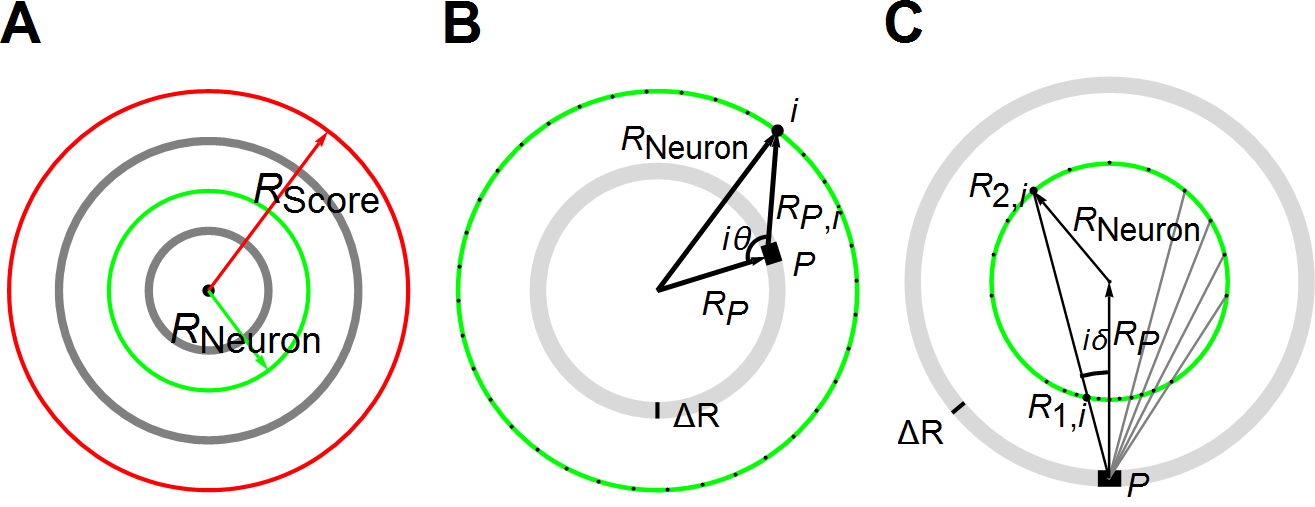

Supplement: S1 Fig — (TIFF) [file pcbi.1004428.s001.tiff]
