# Supplementary material for: Irradiation of Neurons with High-Energy Charged Particles: An In Silico Modeling Approach
Source: PLoS Comput Biol. 2015 Aug 7;11(8):e1004428. doi: 10.1371/journal.pcbi.1004428 (PMC4529238; doi:10.1371/journal.pcbi.1004428)
Supplement: S1 Text — (DOCX) [file pcbi.1004428.s002.docx]

# **Supporting Information**

## **Sensitivity Study of Charged Particle Electronic Equilibrium for Co-Axial Cylinders**

As a consistency check of the scoring methods for energy deposition (ED) to the volume of interest we considered tests of electron equilibrium as described next. A neuron is embedded in a cylindrical volume of *Cyl_Neuron_* with base radius *R_Neuro_*_n_ and axis height, *L_Neuron_*, **Fig. 2A**. The cylinder *Cyl_Neuron_* is embedded in a larger volume cylinder *Cyl_Score_* with larger radius *R_Score_* and axis height *L_Score_*. Both cylinders are aligned along the same axis. A random point (*x_Rand,i_*, *y_Rand,i_* _,_ 0) is chosen at each simulation on the base area of *Cyl_Score_* and a particle beam impinges perpendicular to the base area propagating along the cylinder +*z*-axis. Electrons produced in the medium are scattered radially and longitudinally much larger than the volume of the *Cyl_Score_* and the measurement volume *Cyl_Neuron_* can confine.

The approach of sending the particles on a larger base surface of *Cyl_Score_* and measuring the dose centrally in smaller *Cyl_Neuron_* could sample all the ED voxels created by the particle if the *R_Score_* ˗ *R_Neuron_* is on the order of the distance where radial component of normalized ED reaches unity in **Fig. 5**. The extent of finite particle track length along the propagation direction should still cover longitudinal buffer lengths from the points of *Cyl_Neuron_* base points on forward and backward distances for forward and backward scattering. Then, the voxel ED spectrum in *Cyl_Neuron_* would be a true representation of the all the voxels such that electronic equilibrium would be achieved in a smaller test volume. The distances *L_Forward_* and *L_Backward_* in **Fig. 2A**, along the beam propagation axes are taken to account for scattered electrons in the forward and backward directions giving contributions to the dose of *Cyl_Neuron_*. The axis height *L_Score_* of *Cyl_Score_* is equal to *L_Forward_*+*L_Neuron_*+*L_Backward_*. Likewise, the larger base diameter, 2×*R_Score_*, than the maximum axial range of the neuron (2×*R_Neuron_*) in *Cyl_Neuron_* contributes to the radial dose by scattered particles. The radius, *R_Score_* of *Cyl_Scor_*_e_ is equal to *R_Gap_*+*R_Neuron_* where the *R_Gap_* is the extra axial distance to *Cyl_Neuron_* in **S1 Table**.

To quantify energy deposition bounded by the cylindrical boundaries *L_Neuron_*, and *R_Neuron_*, a geometric kernel approach is followed by; first by creating radial and longitudinal cumulative distribution functions for the nearly mono energetic beams. A large number of histories stored in an irradiation library are generated by the RITRACKs software for selected particles and energies for track length *L_Track_* (20 µm). Each voxel coordinates of each history data and its ED value are recorded in Cartesian coordinates in space by overlapping the histories along the propagation direction starting at (0,0,0) and ending at (0, 0, *L_track_*) for (^56^Fe, ^12^C, ^1^H, e^-^) beams at (600 MeV/u, 300 MeV/u, 250 MeV, (500-to-490) keV), respectively.

A histogram of bin size ($\Delta l=$100 nm) for longitudinal extension of the ED events along the *z*-axis is plotted by locating the voxel values (*x_i_*, *y_i_*, *z_i_*) with their *z_i_* coordinate values. Then, a normalized cumulative total ED function along the *z*-axis is created for further numerical evaluation and this kernel is called the longitudinal cumulative distribution function (LCDF).

A similar approach is followed for radial extension of voxel coordinates and their deposited (voxel) energy values. Axial distance ($r_{ax,i}$) of each voxel coordinate (*x_i_*, *y_i_*, *z_i_*) is calculated as $r_{ax,i}=\sqrt{x_{i}^{2}+y_{i}^{2}}$ and placed in bins of size ($\Delta r=$100 nm) from the $r_{ax}=0$ point. A normalized cumulative histogram data of the bin energies are plotted along the $r$-axis where $r_{ax}=0$ is the beam axial entrance point and this kernel is called the radial cumulative distribution function (RCDF), first column in Fig. 2, in the text.

The LCDF and RCDF numerical functions in **Fig. 4** quantify the extent of longitudinal and radial primary and secondary energy deposition events of particle beams of length *L_Track_*. The next step is to find numerically the dose the target cylinder *Cyl_Neuron_* receives given the fluence to the cylinder *Cyl_Score_* where fluence of the *N* particle beams is uniformly distributed on the base of the cylinder with radius *R_Score_*. Particle beam density, ρ is defined as ρ=$N/{\pi R_{Score}^{2}}$ where *N* is the number of beams and $\pi R_{Score}^{2}$ is the irradiated base area in the simulation.

The radial contribution of dose to the inner embedded cylinder, *Cyl_Neuron_*, is calculated by two components in **S1. Fig A**, i) the beams impinging on the inner area Δ$R_{Neuron}^{2}$ but some ratio of it is scattered out of the area, and ii) the beams at *R_Neuron_*< *r* <*R_Score_* giving contribution by scattered dose to the inner cylinder with *R_Neuron_*.

## **1) Radial contribution of dose to *Cyl_Neuron_* of fluence at** $\boldsymbol{r<}\boldsymbol{R}_{\boldsymbol{Neuron}}$

The method to find the radial contribution of ED to the inner cylinder of radius *R_Neuron_* located centrally inside a cylinder with larger cylinder radius *R_Score_* is first to draw an annulus of small thickness of $\Delta R$. The total number of annuli in *Cyl_Score_* and *Cyl_Neuron_* are given by $m={R_{Score}}/{\Delta r}$ and $d={R_{Neuron}}/{\Delta r}$ where $m$ and $d$ are integers. The annulus is circularly symmetric and the number of beams in the annulus is $\rho2\pi\Delta R{(R}_{p}-{\Delta r}/2)$ and *R_P_* is the annulus radius. For the inner annulus with radius *R_P_* (*R_P_* $<$*R_Neuron_*) in **S1 Fig B**, the contribution of dose from a small patch at point $P$ to the area $\pi R_{Neuron}^{2}$ can be linearly added over whole annulus area. So, the contribution of the annulus at *R_P_* can be found by a mean value of the patch at $P$ for the circularly symmetric annulus. The mean (RCDF(*R_P_*))*_In_* for the inner annulus is numerically calculated over discrete small azimuthal angles $i\times\theta$ from 0 to $2\pi$ at $n$ steps ($\theta=2\pi/n$) from the point $P$ to points *i*’s on the circumference of *R_Neuron_* in **S1 Fig B**.

$$mean\left( RCDF\left( R_{P} \right) \right)_{In}=\frac{1}{n}\sum_{i=1}^{n} RCDF(R_{P,i})$$

RCDF(*R_P,i_*) is the ratio of energy confined to area defined by *R_Neuron_* for a distance *R_P,i_* that is given by $R_{P,i}=R_{P}\cos\left( i\theta\right)+\sqrt{R_{P}^{2}\left( -1+\left( \cos\left( i\theta\right) \right)^{2} \right)+R_{Neuron}^{2}}$ where $\theta={2\pi}/n$. The term for the contribution of full inner cylinder, *radCont_In_*, is given by summing over all the annuli ED contributions as,

$${radCont}_{In} \propto\rho2\pi\Delta R\sum_{P=1}^{d} \left( R_{p}-\frac{\Delta R}{2} \right)\times mean\left( RCDF\left( R_{P} \right) \right)_{In}$$

And for dose bounded by the area *R_Neuron_* ($d\times\Delta r$) *radCont_In_* is simplified to

$${radCont}_{In}=N\frac{2}{d^{2}}\sum_{P=1}^{d} \left( P-\frac{1}{2} \right)\times mean\left( RCDF\left( {\frac{P}{d}R}_{Neuron} \right) \right)_{In}$$

**
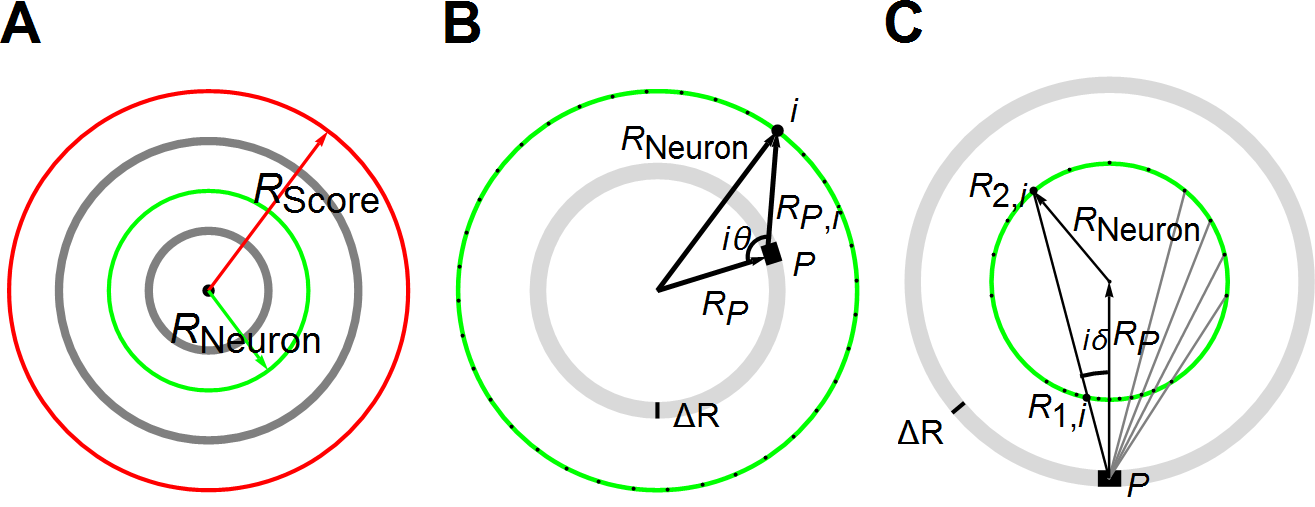
**

**S1 Fig. Radial contribution of dose to inner, *Cyl_Neuron_* volume (A) by particle fluence on *Cyl_Neuron_* (B) and *Cy_lScore_* (C) surface sections.** In the top view, dose to the inner *Cyl_Neuron_*, shown with green circumference circle of radius *R_Neuron_* is calculated by uniform density fluence, $\rho$, impinging on the area bounded by *Cyl_Score_* drawn with red circumference circle of radius *R_Score_* (A). Dose contribution to *Cyl_Neuron_* is split into beams impinging on the inner radius $r<R_{Neuron}$ (B), and outer radius *R_Neuron_*$<r<$*R_Score_* in (C). A small patch, $P$, in black on the circularly symmetric annuli in gray is treated as an infinitesimal source of particle fluence. Numerically calculated, normalized radial deposited energy plots (see main text) determine how much of the deposited energy stays in the *Cyl_Neuron_*. If the source patch is within the *Cyl_Neuron_* with $r<R_{Neuron}$ as in (B), then the mean energy contribution is calculated for each point *i* on the *R_Neuron_* circumference by reading the radial deposited energy (RCDF) values at *R_P,i_* in Fig. 2. RCDF(*R_P,i_*) is the numerical value of source at $r=0$ to $r=R_{P,i}$. If the source patch, $P$, is outside the area of concern, then scattered energy from the source contributes to dose in *Cyl_Neuron_* as in (C), RCDF(*R_2,i_*)˗RCDF(*R_1,i_*) is proportional to how much scattered energy in an a small angle, δ in the text, contributes to the dose in *Cyl_Neuron_*.

## **2) Radial contribution of dose to *Cyl_Neuron_* of fluence at *R_Neuron_***$\boldsymbol{<r<}$***R_Score_***

The contribution of radial component of scattered dose to $\pi R_{Neuron}^{2}$ from the annulus at *R_P_* (*R_Neuron_*$<$*R_P_*$<$*R_Score_*) in **S1 Fig C** can be similarly calculated by a patch at point $P$ on the annulus of small thickness $\Delta R$. $\alpha_{P,max}$ is the maximum radial angle that the point $P$ can contribute to area $\pi R_{Neuron}^{2}$ and is given by $\alpha_{P,max}=2ArcSin(R_{Neuron}/R_{P})$. So, $\alpha_{P.max}/2\pi$ is a scaling factor updated at each *R_P_* for some mean value that will be calculated on the inner area $\pi R_{Neuron}^{2}$. A mean value of scattered energy bounded by the inner circular area can be found by drawing straight lines from point $P$ to the inner area with equal angular intervals and taking the difference between RCDF(*R_2,i_*) and RCDF(*R_1,i_*) where *R_2,i_* and *R_1,i_* are the distances for line *i* between the farther and closer intersection points between the perimeter of the circle and the line emanating from $P$. The difference RCDF(*R_2,i_*) and RCDF(*R_1,i_*) is a representation of the ratio of how much energy is deposited to the area along the line. α_max_ at point $P$ can be divided to $s$ pieces where $\delta={\alpha_{max}}/s$ and a mean value that can be calculated over all the points on the annulus defined by the angle $i\delta$ where $i=1,2,\ldots,s/2$ that is measured between the distances in **S1 Fig C**.

$$mean\left( RCDF\left( R_{P} \right) \right)_{Out}=\frac{\alpha_{P,max}}{2\pi}\frac{1}{s}\sum_{i=1}^{S} RCDF\left( R_{2,i} \right)-RCDF\left( R_{1,i} \right)$$

where *R_1,i_* and *R_2,i_* can be given as

$$R_{1,i}=R_{P}\cos\left( i\delta\right)-\sqrt{R_{P}^{2}\left( -1+\left( \cos\left( i\delta\right) \right)^{2} \right)+R_{Neuron}^{2}}$$

$$R_{2,i}=R_{P}\cos\left( i\delta\right)+\sqrt{R_{P}^{2}\left( -1+\left( \cos\left( i\delta\right) \right)^{2} \right)+R_{Neuron}^{2}}$$

The total contribution from outside to the inner cylinder, *radCont_Out_*, is given by

$${radCont}_{Out}=\rho2\pi\Delta R\sum_{P=d+1}^{m} \left( R_{p}-\frac{\Delta R}{2} \right)\times mean\left( RCDF\left( R_{P} \right) \right)_{Out}$$

which can be simplified to

$${radCont}_{Out}=N\frac{2}{m^{2}}\sum_{P=d+1}^{m} \left( P-\frac{1}{2} \right)\times mean\left( RCDF\left( R_{P} \right) \right)_{Out}$$

The total radial contribution, *radCont* is given by,

$$radCont={radCont}_{In}+{radCont}_{Out}$$

## **Longitudinal Contribution**

A similar methodology is used by utilizing the longitudinal cumulative distribution function of beams of length *L_track_* and calculating the contribution of each beam along the propagation direction bounded by the axis length *L_Neuron_* of the inner cylinder. A finite length simulated beam is composed of linearly added track beams with *L_track_* that goes through the cylinder volume perpendicular to the surface. Discrete number of beams, *N_History_*, to cover the total axis length *L_Score_* and corresponding distances, *L_Forward_*, *L_Backward_* and *L_Neuron_* were chosen in this study (*L_Forward_* and *L_Backward_* were chosen different values for each particle and are given in S1 Table). As discussed above the longitudinal range, *L_Neuron,i_* was variable at each trial and *L_Neuron_*$=$140 µm is taken as the range to ensure a constant value in this calculation between the minimum thickness of the neuron 35 µm and maximum diagonal distance 256 µm.

The start point of a track is plotted as the $z=0$ point on the LCDF plot in S2 Fig. Contribution of each track in a beam to the gray shaded longitudinal volume is calculated by how much of LCDF(*L_k_*) contributes to the volume of interest where *L_i_* are the distances of zero point of the track to the entrance (*L_1_*) and exit point (*L_2_*) distance of the volume of interest (dashed lines in S2 Fig). To secure a positive value of dose in the volume of interest the difference of LCDF(*L_k_*) ($k=1,2$) values expressed as an absolute valued quantity. Basically, the Abs(LCDF(*L_2_*) ˗ LCDF(*L_1_*)) is the contribution of track of interest to the volume of interest in the longitudinal direction.

Contributions from all the track segments are summed and normalized by the *N_z_* that is the number of tracks per inner cylinder axis length; $N_{z}={L_{Neuron}}/{L_{track}}$. If fractional number of beams is arranged along the *L_Neuron_* the same argument of counting *N_z_* and calculating the contribution of tracks hold true.

$$longCont=\frac{1}{N_{z}}\sum_{i=1}^{all} Abs(LCDF\left( L_{2,i} \right)-LCDF\left( L_{1,i} \right))$$

**Figure 3** depicts the longitudinal component of dose contribution to the inner, *Cyl_Neuron_* cylinder section. The contributions of the tracks (13 in this figure) are added and the sum is divided by the number of tracks bounded by the length *L_Neuron_* (8 in the figure). In the example of **Fig. 3**, the contributions of the three red tracks are found as; 1^st^: 0.0746 (0.9742˗0.8996); 2^nd^: 0.9536 (0.9577˗0.0041), and 3^rd^: 0.0030 (0.0082˗0.0052). The tracks along both *L_Forward_* and *L_Backward_* act as the longitudinal component of build-up materials to reach electronic equilibrium in *L_Neuron_*.

The overall electronic equilibrium factor (*Elec.Equi.Fac.*) is calculated by multiplying the independent factors *radCont* and *longCont* values;

$$Elec.Equi.Fac.=radCont\times longCont$$

The corresponding parameters are given in **S1 Table** where *PredictedDose* is

$$PredictedDose=FluenceDose\times Elec.Equi.Fac.$$

**Table A. Calculated dose values and the geometric parameters used in this study.**

|  | *radCont* | *longCont* | *Elec.Eq.*  *Fac.* | *Fluence*  *Dose*(Gy) | *Predicted*  *Dose*(Gy) | *Neuron*  *Dose*(Gy) | *R_Gap_*  (µm) | *L_Forward_*  (µm) | *L_Backward_*  (µm) |
| --- | --- | --- | --- | --- | --- | --- | --- | --- | --- |
| ^56^F | 0.916 | 0.957 | 0.876 | 2.27 | 1.99 | 1.79 | 150 | 120 | 20 |
| ^12^C | 0.937 | 0.971 | 0.910 | 2.64 | 2.40 | 2.31 | 190 | 100 | 60 |
| ^1^H | 0.956 | 0.963 | 0.920 | 1.34 | 1.24 | 1.16 | 50 | 20 | 20 |
| e^-^ | 0.934 | 0.742^a^ | 0.693 | 1.30 | 0.90 | 0.92 | 190 | 80 | 160 |

- PredictedDose is calculated for an average value of *R_Neuron_*=128 µm, *L_Neuron_*=140 µm.
- a) Average *L_Track_*=46.4 µm is taken to calculate *longCont* in agreement with the algorithm to ensemble of an electron beam.

The test neuron is filling only a small portion of the *Cyl_Neuron_* (~0.039%) for *L_Neuron_*= 140 µm and the neuron configuration is changing at each trial. The estimated value of electronic equilibrium approach may require further study to take into account this variability, especially for very high energy particles.
